# Magnesium biology

**DOI:** 10.1093/ndt/gfae134

**Published:** 2024-06-13

**Authors:** Jana L Kröse, Jeroen H F de Baaij

**Affiliations:** Department of Medical BioSciences, Radboudumc, Nijmegen, The Netherlands; Department of Medical BioSciences, Radboudumc, Nijmegen, The Netherlands

**Keywords:** drug-induced hypomagnesemia, hypomagnesemia, magnesium homeostasis, magnesium pathophysiology, SGLT2 inhibitors

## Abstract

Magnesium (Mg^2+^) is essential for energy metabolism, muscle contraction and neurotransmission. As part of the Mg–ATP complex, it is involved in over 600 enzymatic reactions. Serum Mg^2+^ levels are tightly regulated between 0.7 and 1.1 mmol/L by interplay of intestinal absorption and renal excretion. In the small intestine, Mg^2+^ is absorbed paracellularly via claudin-2 and -12. In the colon, transcellular absorption of Mg^2+^ is facilitated by TRPM6/7 and CNNM4. In the kidney, the proximal tubule reabsorbs only 20% of the filtered Mg^2+^. The majority of the filtered Mg^2+^ is reabsorbed in the thick ascending limb, where the lumen-positive transepithelial voltage drives paracellular transport via claudin-16/-19. Fine-tuning of Mg^2+^ reabsorption is achieved in the distal convoluted tubule (DCT). Here, TRPM6/7 tetramers facilitate apical Mg^2+^ uptake, which is hormonally regulated by insulin and epidermal growth factor. Basolateral Mg^2+^ extrusion is Na^+^ dependent and achieved by CNNM2 and/or SLC41A3. Hypomagnesemia (serum Mg^2+^ <0.7 mmol/L) develops when intestinal and/or renal Mg^2+^ (re)absorption is disturbed. Common causes include alcoholism, type 2 diabetes mellitus and the use of pharmacological drugs, such as proton-pump inhibitors, calcineurin inhibitors and thiazide diuretics. Over the last decade, research on rare genetic and acquired Mg^2+^ disorders have identified Mg^2+^ channel and transporter activity, DCT length, mitochondrial function and autoimmunity as mechanisms explaining hypomagnesemia. Classically, treatment of hypomagnesemia depended on oral or intravenous Mg^2+^ supplementation. Recently, prebiotic dietary fibers and sodium-glucose cotransporter 2 inhibitors have been proposed as promising new therapeutic pathways to treat hypomagnesemia.

## INTRODUCTION

### Chemical characteristics

Magnesium is part of the alkaline earth metals in the periodic table, with the atomic number 12 [1]. Magnesium readily forms chemical bonds with phosphate, an essential structure-forming element for DNA, RNA and ATP, which are the underlying building blocks of a multitude of reactions in the human body [2]. Magnesium can only be found in its ionized state (Mg^2+^), bound to proteins, negatively charged molecules and ATP [3]. ATP hydrolyses with Mg^2+^, forming the Mg–ATP complex, which is considered the active form of ATP. This active ATP is capable of facilitating a multitude of cellular and molecular pathways, which are essential for numerous of physiological processes. Next to this, it acts as a co-enzyme for more than 600 enzymatic reactions, including DNA repair mechanisms, nerve impulse propagation, bone formation and many more [1, 2].

### Magnesium in the body

Mg^2+^ shows chemical similarities with calcium (Ca^2+^) and zinc (Zn^2+^). Consequently, their transport in the body is often facilitated via the same divalent cation transporters, channels and pathways [1]. However, Mg^2+^ is considerably larger in its hydrated states. Mg^2+^ binds water (H_2_O) tighter than Ca^2+^, making its dehydration substantially harder, which makes it important for Mg^2+^ transporters to be able to detect hydrated Mg^2+^ and strip it from its hydration shell [4]. Therefore, it is possible for Mg^2+^ transporters to also transport the smaller hydrated Ca^2+^ but it is not possible for the Ca^2+^ transporters to transport the larger hydrated Mg^2+^ [5].

### Magnesium storage and homeostasis in the body

Mg^2+^ is the second most abundant cellular ion and the fourth most abundant ion in the human body. Total body Mg^2+^ accumulates to about 20–28 g in a healthy adult of which 50%–60% is stored in bone, 34%–39% in muscles, soft tissues and organs, and only 1%–2% in blood and extracellular fluids. The reference range for serum Mg^2+^ is between 0.7 and 1.0 mmol/L [6].

Serum Mg^2+^ is present in three forms: about 5%–15% is bound to anions such as phosphate, citrate or bicarbonate, 20%–30% is protein-bound and about 55%–70% of Mg^2+^ is available as ionized Mg^2+^ [3]. Ionized Mg^2+^ is considered the biologically active form of Mg^2+^, taking part in enzymatic reactions and physiological processes. However, in the clinic total serum Mg^2+^ is assessed as measure of Mg^2+^ levels, while assessment of whole-body Mg^2+^ in the form of ionized Mg^2+^ would offer a better representation of the overall Mg^2+^ status.

Recently, discussions about the implementation of a Magnesium Depletion Score (MDS) have been raised. Fan *et al*. (2021) developed the MDS and concluded that it was able to predict the development of hypomagnesemia in individuals even when sex and gender were accounted for in the analysis [7]. However, the MDS does not take factors such as genetic predispositions, autoimmunity or diabetes into account, while they can all play an important role in development of hypomagnesemia. Nevertheless, the MDS presents a valuable tool that can be used as a first screening of patients to identify their risk of developing hypomagnesemia. Additionally, recently Rosanoff *et al*. (2022) suggested uniformalizing cut-offs for hypo-, normo- and hypermagnesemia. Currently, the exact cut-offs for patients to be considered to have hypomagnesemia are highly variable, which ultimately leads to patients being undiagnosed as well as potentially missing symptoms related to hypomagnesemia [8].

Bone and liver have been demonstrated to act as major Mg^2+^ stores in the body. Indeed, *in vitro* and *in vivo* studies showed that osteoclast activity is increased upon decreased extracellular Mg^2+^ concentrations [9, 10]. Moreover, recent data demonstrated that defects in liver Mg^2+^ homeostasis directly affect serum Mg levels [11]. Together these results point towards an involvement of bones and liver in the overall body Mg^2+^ homeostasis [9, 10, 12], which is why there has been a long-running discussion around the ideal determination of whole-body Mg^2+^ levels. Since only about 1%–2% of the whole-body Mg^2+^ is present in serum, it has been suggested that soft tissues and bones can be depleted of Mg^2+^, even if serum Mg^2+^ is normal. However, the clinical relevance of these lower tissue Mg^2+^ contents is unclear.

### Consumption and supplementation of magnesium in health and disease

The daily recommended dietary allowance for Mg^2+^ in adults is about 400 mg for males and 310 mg for females, which increases to 350 mg during pregnancy [13]. According to a report from the European Food Safety Authority combining data from 13 dietary surveys conducted in the European Union, the average Mg^2+^ intake in these studies ranged from 232 to 439 mg/day for adults, which suggests that the majority of the population ingests sufficient Mg^2+^ [14]. Nevertheless, a trend towards the use of Mg^2+^ supplementation can be observed in recent years. Many claim that Mg^2+^ supplementation helps with sleep, muscle cramps and anxiety. Several meta-analyses and reviews conclude that there is not enough scientific evidence to prove that these claims are true in healthy individuals [15–17]. However, Mg^2+^ supplementation can be of therapeutic use in patients suffering from hypomagnesemia [2]. In healthy individuals, increased ingestion of Mg^2+^ poses little risk. In the case of increased ingestion, the intestinal absorption is decreased, while the renal excretion rate can increase up to 100% to balance Mg^2+^ levels. Still, it should be noted that extremely high doses of Mg^2+^ supplementation can lead to symptoms such as diarrhea, nausea and abdominal cramping, but will resolve once the Mg^2+^ has been digested.

## MAGNESIUM HOMEOSTASIS

### Intestinal absorption

**Figure 1:**
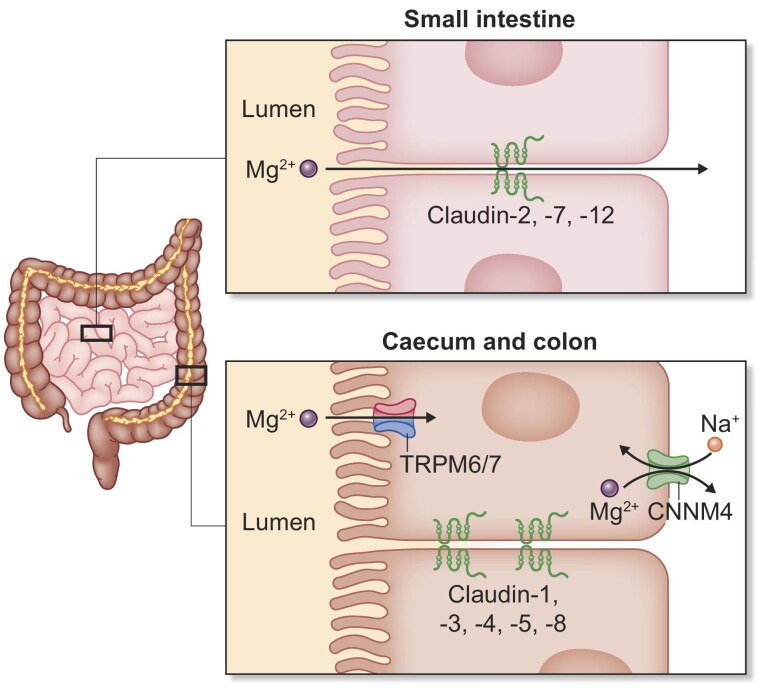
Intestinal magnesium uptake. Intestinal absorption in the small intestines is mainly driven by paracellular transport facilitated by claudin-2, -7 and -12. Luminal Mg concentrations are linearly correlated to Mg^2^^+^ uptake, making the former the driving force of Mg^2^^+^ uptake. In the caecum and colon magnesium is absorbed via the transcellular pathway. Apically, TRPM6/7 is responsible for magnesium influx while CNNM4 as potential sodium–magnesium exchanger facilitates magnesium efflux on the basolateral side. CNNM4 = Cyclin M4; TRPM6/7 = Transient receptor potential family member 6 and 7 heterotetradimers.

In healthy individuals, about 30%–50% of the ingested Mg^2+^ is absorbed in the intestines, which can increase up to 80% during Mg^2+^ deficiency [18]. Mg^2+^ absorption in the intestines is facilitated by either paracellular or transcellular transport, the former accounting for about 90% of the total Mg^2+^ uptake. Looking at the anatomy of the intestinal track, the small intestine—specifically the duodenum and ileum—mainly absorb Mg^2+^ in a paracellular fashion, while the caecum and colon facilitate Mg^2+^ uptake via the transcellular route (Fig. 1). In contrast to the small intestines, tightening claudin-1, -3, -4, -5 and -8 are highly expressed in the colon and lead to reduced paracellular transport. Consequently, transcellular uptake accounts for 30% of Mg^2+^ uptake in the colon, or may even increase further in the presence of low luminal Mg^2+^ [19].

Paracellular Mg^2+^ absorption via the intestinal epithelium is dependent on luminal Mg^2+^ concentration, as this correlates linearly with Mg^2+^ absorption and is facilitated by the tight junctions which regulate ion absorption but also other essential (macro-)nutrients and water [18]. In particular, the expression of claudin-2,-7 and -12 facilitate cation specificity of the intestinal barrier [18, 20–22]. There have been studies suggesting a lumen-negative potential in the small intestines, which theoretically should lead to reduced Mg^2+^ uptake [23]. However, there has been little evidence for this, and further studies are needed.

The transcellular pathway is facilitated by several Mg^2+^ channels and transporters expressed in epithelial cells. Luminal Mg^2+^ absorption is facilitated by a heterotetrametric channel composed of transient receptor potential family member 6 and 7 (TRMP6 and TRPM7) subunits. The selectivity profile of TRPM7 is Zn^2+^ > Mg^2+^ > Ca^2+^. Co-expression with TRPM6 increases its Mg^2+^ permeability, because of the selectivity profile of TRPM6 (Mg^2+^ >Ca^2+^) and reduced inhibition by Mg–ATP [24, 25]. To increase Mg^2+^ absorption, TRPM6 can be stimulated by epidermal growth factor (EGF) and insulin via the PI3K/Akt pathway [18]. Basolateral extrusion of Mg^2+^ is facilitated by Cyclin M4 (CNNM4), which has been suggested to act as a sodium (Na^+^)-Mg^2^^+^ exchanger [26]. CNNM4 is the molecular target of fibroblast growth factor-23 (FGF23) and parathyroid hormone (PTH), which inhibit intestinal Mg^2+^ uptake via protein kinase C [27].

### Renal excretion

Every day the human kidneys filter about 2400 mg of Mg^2+^ of which approximately 95% is reabsorbed along the nephron [1]. While the proximal tubule (PT) facilitates the majority (60%–70%) of Na^+^, potassium (K^+^), chloride (Cl^−^), Ca^2+^ and H_2_O reabsorption, only 20%–30% of filtered Mg^2+^ is reabsorbed in the PT [28]. Although the exact mechanisms of Mg^2+^ transport in the PT have yet to be unraveled, paracellular transport in the late PT is established as the main pathway. Cation-selective claudins-2/-12 have been proposed as the main Mg^2+^ pores in the PT [29]. However, claudin-2 and claudin-12 knock-out (KO) mice did not show hypomagnesemia [30]. Important to consider is that the PT is only responsible for a minor part of the total Mg^2+^ reabsorption in the nephron. Therefore, downstream transport processes may compensate for decreased Mg^2+^ uptake in the PT. Interestingly, a study with claudin-10a KO mice showed increased claudin-2 expression in the tight junctions [31]. Due to a shift in the electrochemical gradient during the knockout of the anion pore claudin-10a, as well as increased selectivity for cation, increased renal Mg^2+^ retention takes place [31].

**Figure 2:**
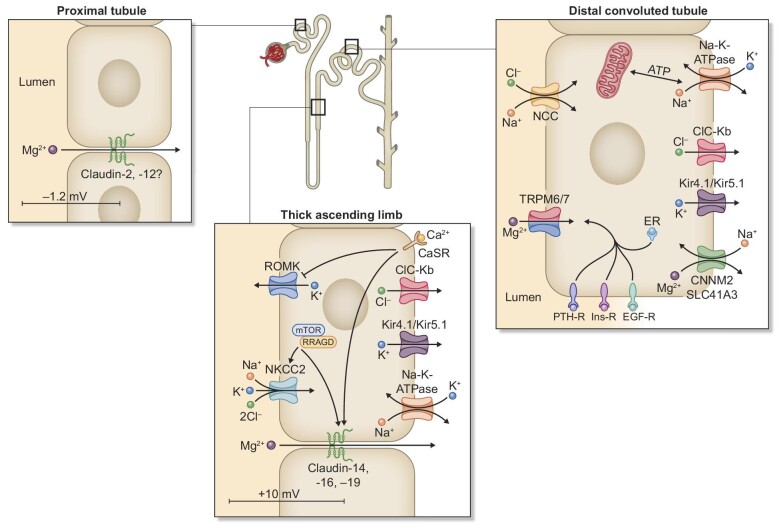
Renal magnesium regulation. Claudin -2 and -12 are thought to be the main key players driving magnesium reabsorption in the PT facilitated by a slightly lumen negative transepithelial voltage. In the TAL, NKCC2 and ROMK are responsible for maintaining a lumen positive transepithelial voltage, mainly through the back leak of potassium into the luminal space. Claudin-14, -16 and -19 are responsible for magnesium reabsorption by paracellular transport. RRAGD activated mTorc1 which is thought to influence NKCC2 and Claudins-14, -16 and -19 leading to increased magnesium uptake. Basolaterally, Na-K-ATPase, Kir4.1/Kir5.1, ClC-Kb and CaSR are responsible to maintain intracellular sodium, potassium and chloride levels in balance. CaSR can be activated by calcium leading to inhibition of ROMK and increased claudin-14 expression, ultimately leading to a change in the transepithelial voltage. In the DCT, magnesium influx is facilitated by the TRPM6/7 channel. TRPM6 is regulated by PTH, insulin, EGF and estrogen. NCC is responsible for sodium and chloride influx. ClC-Kb, Kir4.1/Kir5.1 Na-K-ATPase and CNNM2/SLC41A3 are located on the basolateral side. The role of CNNM2 in magnesium homeostasis is heavily discussed, however it has been suggested that CNNM2 acts as a sodium–magnesium exchanger. Since the debate has not been settled yet, potential other sodium–magnesium exchangers haven been explored further, one being SLC41A3. CaSR = Ca^2^^+^ sensing receptor; CNNM2 = Cyclin M2; ClC-Kb = voltage-gated Cl^–^ channel Kb; EGF-R = EGF receptor; ER = estrogen receptor; Ins-R = insulin receptor; Kir = inward rectifier-type K^+^ channel; NCC = Na^+^-K^+^ cotransporter; NKCC2 = Na^+^-K^+^-2Cl- cotransporter; PTH-R = PTH receptor; ROMK = renal outer medullary K^+^ channel; TRPM6/7 = Transient receptor potential family member 6 and 7 heterotetradimers.

The thick ascending limb of Henle's loop (TAL) is responsible for 50%–70% of renal Mg^2+^ reabsorption. Mg^2+^ reabsorption in the TAL is facilitated via paracellular transport driven by the lumen-positive transepithelial voltage created by the combined action of the Na^+^-K^+^-2Cl^−^ co transporter (NKCC2), Na^+^-K^+^-ATPase, renal outer medullary K^+^ channel (ROMK), inward rectifier-type K^+^ channel (Kir4.1/5.1) and voltage-gated Cl^−^ channel (ClC-Kb) [32] (Fig. 2). Mutations in *CLDN16* and *CLDN19* cause familial hypomagnesemia with hypercalciuria and nephrocalcinosis (FHHNC), demonstrating their involvement in Mg^2+^ reabsorption [33, 34]. *In vitro* studies using perfused TAL tubules showed that Mg^2+^ permeability was decreased after deletion of claudin-16. Additionally, Madin–Darby canine kidney (MDCK) cell models with overexpression of claudin-16 showed increased Mg^2+^ flux, while overexpression of claudin-19 in MDCK cells resulted in decreased Mg^2+^ flux [35, 36]. Moreover, claudin-16 and claudin-19 KO mice display decreased serum Mg^2+^ levels [37, 38]. Altogether, these results allow to conclude that claudin-16 and claudin-19 are responsible for paracellular Mg^2+^ transport. In contrast, claudin-14 blocks paracellular transport [39]. Claudin-14 expression is upregulated by Ca^2+^ sensing receptor (CaSR) activation, creating a feedback loop in which increased basolateral activation of CaSR increases claudin-14, resulting in decreased paracellular uptake [39, 40].

**Figure 3:**
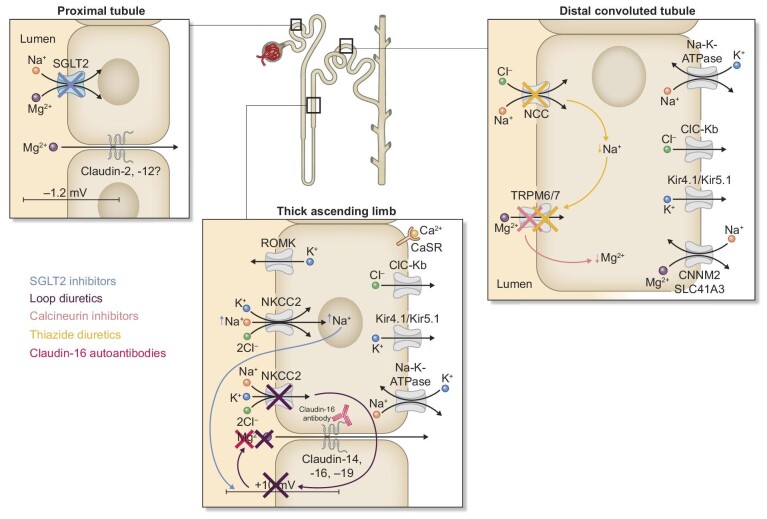
Mechanisms of drug-induced-hypomagnesemia and autoimmunity Claudin-16 autoantibodies are directed against Claudin-16 resulting in misfunction and decreased magnesium reabsorption in the TAL. Loop diuretics act in the TAL by inhibition of NKCC2 resulting in a disturbance of the transepithelial membrane potential resulting in disruption on magnesium reabsorption. The opposing process is thought to take place during the use of SGLT2 inhibitors. Increased sodium levels in the preurine lead to overactivation of NKCC2 resulting in an increased transepithelial membrane potential driving magnesium reabsorption. Thiazide diuretics block NCC in the DCT leading to decreased sodium levels resulting in inhibition of TRPM6/7. Calcineurin inhibitors lead to decreased expression of TRPM6 resulting in reduced Mg^2^^+^ uptake in the DCT. CaSR = Ca^2^^+^ sensing receptor; CNNM2 = Cyclin M2; ClC-Kb = voltage-gated Cl^–^ channel; EGF-R = EGF receptor; ER = estrogen receptor; Ins-R = insulin receptor; Kir = inward rectifier-type K^+^ channel; NCC = Na^+^-K^+^ cotransporter; NKCC2 = Na^+^-K^+^-2Cl^–^ cotransporter; PTH-R = PTH receptor; ROMK = renal outer medullary K^+^ channel; TRPM6/7 = Transient receptor potential family member 6 and 7 heterotetradimers; SGLT2 = sodium-glucose cotransporter 2.

The distal convoluted tubule (DCT) is responsible for reabsorption of 5%–10% of the filtered Mg^2+^. Luminal Mg^2+^ uptake in DCT cells is facilitated via TRPM6 and TRPM7 heterotetradimers. Although TRPM6 requires TRPM7 to be functional, its expression is essential as TRPM6–TRPM7 tetramers have an increased Mg^2+^ transport capacity compared with TRPM7 homomers [41]. However, the exact role of TRPM7 in this segment still has to be elucidated. Recently, pathogenic variants of TRPM7 have been linked to hypomagnesemia, indicating its role in Mg^2+^ reabsorption [42]. TRPM6 activity is stimulated by insulin, EGF, PTH and estrogen, and inhibited by increased intracellular Mg^2+^ concentrations [43]. Cyclin M2 (CNNM2) has been implicated in basolateral Mg^2+^ extrusion. The exact mechanistic function of CNNM2 has been under discussion for years. Some groups claim that CNNM2 acts an Na^+^-Mg^2^^+^ exchanger, while other suggest that CNNM2 is a regulatory protein involved in Mg^2^^+^ sensing [26, 44]. Recent studies confirmed the interaction of CNNM2 with TRPM7 in a larger complex of regulatory proteins, including phosphate of regenerating liver (PRL-1/2) and ADP-ribosylation factor-like15 (ARL-15) [45]. However, these studies have not been performed in polarized kidney cells and therefore, the relevance of these findings for the DCT remain unclear. Due to the controversy surrounding the function of CNNM2, other potential Na^+^-Mg^2^^+^ exchangers have been proposed as basolateral Mg^2+^ extrusion mechanisms. SLC41A1 has been extensively studied *in vitro* and *in vivo.* However, SLC41A1 KO mice have normal serum Mg^2+^ levels. SLC41A3 on the other hand, is highly expressed in the DCT and *in vivo* knockout models show a hypomagnesemia phenotype [46].

## MAGNESIUM PATHOPHYSIOLOGY

Mg^2+^ deficiency (serum Mg^2+^ <0.7 mmol/L) can result in symptoms such as fatigue, muscle cramps and arrythmias, and in severe cases developmental delay and seizures [2]. Risk factors for the development of hypomagnesemia include age, gastrointestinal track diseases, type 2 diabetes and excessive alcohol consumption, as well as the use of certain pharmacological drugs [47, 48]. With a prevalence of ±2.5% in the general population and about 12%–20% in hospitalized patients, hypomagnesemia is especially important in patients with pre-existing comorbidities, and needs to be treated adequately [48].

Patients rarely present with hypermagnesemia, which can be caused by excessive amount of Mg^2+^ ingestion either through Mg^2+^ supplementation or drugs high in Mg^2+^ such as laxatives and Epsom salts [49, 50].

Notably, hypomagnesemia often is accompanied by hypokalemia or hypocalcemia. Hypokalemia is caused by decreased inhibition of ROMK in the collecting duct in response to hypomagnesemia. ROMK is inhibited by Mg^2+^, stimulating basolateral excretion of K^+^. Therefore, low Mg^2+^ levels lead to decreased inhibition of ROMK, resulting in increased efflux of K^+^ into the pre-urine resulting in hypokalemia and increased excretion of K^+^ [51].

Low Mg^2+^ levels lead to increases CaSR signaling in the parathyroid gland, resulting in decreased PTH secretion ultimately resulting in decreased Ca^2+^ levels via secondary mechanisms such as decreased vitamin D levels needed for Ca^2+^ absorption [52]. However, the exact mechanism and regulation of Ca^2+^ levels in response to hypomagnesemia remain elusive.

### Alcohol-induced hypomagnesemia

Hypomagnesemia is often seen in people with increased alcohol consumption. It has already been shown decades ago that chronic alcohol consumption is associated with decreased serum Mg^2+^ levels and hypermagnesuria [53]. Next to the direct effect of alcohol on urinary Mg^2+^ wasting, secondary effects such as vomiting or diarrhea may further contribute to hypomagnesemia in alcohol-dependent patients. Furthermore, other comorbidities of alcohol abuse, such as acute pancreatitis and cirrhosis, have been shown to be associated with decreased serum Mg^2+^ levels [54–56]. Mg^2+^ supplementation has been suggested to ease alcohol withdrawal, while it is remains inconclusive whether Mg^2+^ positively influences this process [57]. A recent study demonstrated that 60% of patients with alcohol withdrawal syndrome have hypomagnesemia, which was associated with an increased 1-year mortality risk [58].

### Diabetes-induced hypomagnesemia

One of the most common causes of hypomagnesemia is type 2 diabetes mellitus (T2DM). Countless observational cohort studies have established an inverse association between T2DM and serum Mg^2+^ levels as well as Mg^2+^ intake [59]. The incidence rate of hypomagnesemia in diabetes patients ranges from 10% to 45% [60, 61]. T2DM patients often present with hypermagnesuria, indicating disturbed renal Mg^2+^ handling as the underlying cause of hypomagnesemia [62]. Clinical trials investigating the effect of Mg^2+^ supplementation show conflicting results. Some studies show a minor improvements of fasting plasma glucose levels, HOMA-IR and triglyceride levels in the blood, while other did not observe a beneficial effect of Mg^2+^ supplementation on these factors in T2DM patients [63–65]. It has also been shown that Mg^2+^ can be bound to free fatty acids (FFA), which makes Mg^2+^ undetectable with tests used for normal magnesium assessment. This results in the question of whether Mg^2+^ levels are decreased in T2DM patients or if the Mg^2+^ present, is bound to FFA [66]. Another potential parameter in the inconclusive results could be the difficulty to obtain a substantial increase in serum Mg^2+^ levels via oral supplementation. If there is no substantial increase, then it is difficult to make conclusive statements about the effects of Mg^2+^ on T2DM. However, the relationship between T2DM and Mg^2+^ remains to be further investigated, and in particular aspects such as the influence of magnesium binding to FFA and increase in serum Mg^2+^ levels are of interest.

### Drug-induced hypomagnesemia

Drug-induced hypomagnesemia can be caused by a multitude of drugs [67]. In the following paragraphs, we will focus on the most common causes of drug-induced hypomagnesemia: protein pump inhibitors (PPIs), immunosuppressive drugs and diuretics (Fig. 3) [47]. A full overview of all drugs associated with hypomagnesemia has been provided by Bosman *et al*. (2021) [67].

PPIs are commonly used to treat reflux disease, peptic ulcers, esophagitis, Zollinger–Ellison syndrome and several other gastric acid–related diseases. PPIs inhibit the gastric H^+^/K^+^-ATPase and are commonly used in the general population (10%–20% in the Western world) [68]. Although PPIs have initially been celebrated because of their excellent safety profile, over the last decade prolonged PPI use has been associated with hypomagnesemia. A meta-analysis demonstrates that especially the duration and dosage of the PPIs is a strong indicator for the development of hypomagnesemia [69]. Different mechanisms of how PPIs cause hypomagnesemia have been proposed [20]. Inhibition of acid secretion in the stomach has been shown to increase the pH levels in the small intestine and colon. Increased pH levels decrease the solubility of Mg^2+^, leading to reduced paracellular Mg^2+^ absorption in the small intestine. Additionally, TRPM6 activity is decreased at higher pH levels and the colonic microbiome composition is disturbed, both of which may contribute to decreased Mg^2+^ uptake in the colon [20].

Several immunosuppressive drugs interfere with renal Mg^2+^ reabsorption. In particular, calcineurin inhibitors (CNIs) are strongly associated with severely decreased serum Mg^2+^ levels and are generally recognized as nephrotoxic [70]. CNIs lead to decreased expression of TRPM6, consequently leading to hypomagnesemia [71].

Diuretics are antihypertensive drugs and are commonly used for volume overload conditions such as nephrotic syndrome, congestive heart failure and cirrhosis [47]. In particular, thiazide diuretics are associated with a decrease in serum Mg^2+^ level of 5%–10% [47]. The effects of diuretics on Mg^2+^ level have been well established *in vitro*, and *in vivo* in animal studies. Loop diuretics negatively impact Mg^2+^ transport in the TAL by inhibition of NKCC2, disrupting the lumen-positive transmembrane potential usually driving reabsorption of Mg^2+^. *In vitro* models showed that this paracellular Mg^2+^ transport is disrupted after administration of loop diuretics [72]. Additionally, some but not all patients receiving loop diuretics show hypomagnesuria, which is strongly dependent on the dose and duration of use [73]. A double-blind placebo-controlled study showed that use of loop diuretics lead to increased Mg^2+^ and Ca^2+^ excretion after 3 h, while after 24 h the urinary excretion of these ions was decreased, indicating possible compensatory mechanisms [73, 74]. Thiazide diuretics block NCC, decreasing the intracellular sodium level in the DCT, which indirectly leads to inhibition of TRPM6/7 resulting in decreased Mg^2+^ uptake in the DCT. It has been observed *in vivo* that blocking of NCC leads to a decrease in DCT length as well as inhibition of TRPM6 via secondary mechanisms, resulting in impaired Mg^2+^ reabsorption [75, 76]. However, hypomagnesemia as a consequence of diuretic use is seen comparably seldom in patients using diuretics [77]. Potentially, there is a comorbidity present in some patients, increasing the risk for hypomagnesemia during diuretic use.

### Autoimmunity and hypomagnesemia

Recently, autoimmunity has been proposed as novel disease-mechanism in a patient with hypomagnesemia. Figueres *et al*. (2022) reported an adult male patient with acquired hypomagnesemia, hypocalcemia and tubulointestinal nephropathy with rapidly progressing kidney injury [78]. The presence of claudin-16 autoantibodies was confirmed via immunostaining of mouse kidney and further investigated in mouse kidney TAL (MKTAL) cells. Mouse kidney tissue was incubated once with patient plasma and once with control plasma and stained with human immunoglobulin G (IgG). Sections incubated with patient plasma showed immunofluorescence with human IgG staining, while the control condition did not show this. This indicates the presence of autoantibodies in patient plasma [78]. Furthermore, patient IgG and control IgG from healthy donors was intravenously injected into Sprague–Dawley rats, leading to increased renal fractional excretion of Mg^2+^ at Day 3 post-injection [78]. Together, these findings suggest autoantibodies as a new cause for renal Mg^2+^ wasting. However, the prevalence of autoimmunity as cause for acquired hypomagnesemia remains to be examined.

### Genetic hypomagnesemia

In the last two decades, several hereditary causes of hypomagnesemia have been identified. Although it goes beyond the scope of this review to describe each individual gene, the genetic causes of hypomagnesemia can be grouped by their disease mechanisms. Mutations affecting the paracellular Mg^2+^ transport pathway in the TAL (*CLDN16, CLDN19, RRAGD, CASR*) results in the hypomagnesemia with hypercalciuria and nephrocalcinosis [79]. In the DCT, mutations in *TRPM6* or *TRPM7* cause hypomagnesemia with secondary hypocalcemia. Additionally, patients with disturbed Na^+^ reabsorption in the DCT present with a Gitelman-like syndrome, characterized by hypokalemia, hypomagnesemia and metabolic alkalosis (*SLC12A3, CLCNKB, KCNJ10, KCNJ16, ATP1A1, FXYD2, HNF1B, PCBD1*). In recent years, hypomagnesemia has also been described in patients with mitochondrial disorders (*MT-TI, MT-TF, SARS2* and others) [80]. Other causes of isolated hypomagnesemia include *KCNA1, EGF* and *CNNM2*. Although the mechanisms that explain the interplay between Na^+^ and Mg^2+^ reabsorption in the DCT are only partially understood, decreased DCT length seems to contribute to Mg^2+^ wasting in Gitelman-like syndromes. Mutations in *CLDN10* have been associated with hypermagnesemia, while *in vivo* studies in CLDN10 KO mice resulted in hypermagnesemia [81, 82].

All genetic causes of hypomagnesemia have been recently reviewed by de Baaij *et al*. (2023) and have been summarized in Table 1 [29].

**Table 1:** Genetic causes of disturbed magnesium homeostasis.

Segment	Gene	Protein	Other symptoms	Disease	Serum Mg^2+^	Serum Ca^2^^+^	Serum K^+^
TAL	CLDN10	Claudin-10	Hypohidrosis, lacrimal gland dysfunction	Helix	+	=	–
	CLDN16	Claudin-16	Chronic kidney disease, nephrocalcinosis	FHHNC type 1	–	=	=
	CLDN19	Claudin-19	Chronic kidney disease, nephrocalcinosis	FHHNC type 2	–	=	=
	CaSR	CaSR	Chronic kidney disease, nephrocalcinosis	Autosomal dominant hypocalcemia	–	+	–
TAL?/DCT?	FAM111A	FAMA111A		Kenny Caffey syndrome type 2	–	–	=
TAL/DCT	RRAGD	RagD	Dilated cardiomyopathy	Autosomal dominant hypomagnesemia—RRAGD	–	=	=
	CLCKNB	ClC-Kb		Bartter type 3	–/=	=	–
DCT	SLC12A3	NCC	Chondrocalcinosis	Gitelman	–	=	–
	MT-TI	Mitochondrial-tRNA-IIe		Mitochondrial Gitelman	–	=	–
	MT-TF	Mitochondrial-tRNA-Phe	Chronic kidney disease	Mitochondrial Gitelman	–	=	–
	TRPM6	TRPM6		HSH type 1	–	–	=
	TRPM7	TRPM7		HSH type 2	–	–/=	=
	KCNJ10	Kir4.1	Deafness, ataxia, intellectual disability	Epilepsy, ataxia, sensorineural deafness and tubulopathy (EAST)	–	=	–
	KCNJ16	Kir5.1	Deafness	Hypokalemic tubulopathy and deafness	–/=	=	–
	EGF	EGF	Intellectual disability	Autosomal recessive hypomagnesemia—EGF	–	=	=
	KCNA1	Kv1.1	Ataxia, myokymia	Autosomal dominant hypomagnesemia—KCNA1	–	=	=
	ATP1A1	α-Subunit of Na^+^-K^+^-ATPase	Intellectual disability	Autosomal dominant hypomagnesemia—ATP1A1	–	=	–/=
	FXYD2	γ-Subunit of Na^+^-K^+^-ATPase		Autosomal dominant hypomagnesemia—FXYD2	–	=	–/=
	HNF1B	HNF1β	Chronic kidney disease, renal cysts, mature-onset diabetes of the young	Autosomal dominant tubulointerstitial kidney disease—HNF1B	–	=	–/=
	PCBD1	PCBD1	Mature-onset diabetes of the young	Hyperphenylalaninemia, BH_4_-deficient	–	=	=
	CNNM2	CNNM2	Intellectual disability, obesity	Hypomagnesemia, seizures, and mental retardation	–	–/=	=

+ indicates increased serum levels, = indicates stable serum levels, – indicates decreased serum levels.

### Therapeutics for magnesium disorders

Classic treatment of hypomagnesemia relies on Mg^2+^ supplementation, either orally or intravenously depending on the severity of the disease [2]. Moreover, clinicians may treat common underlying causes, such as alcoholism and diabetes mellitus, or halt the prescription of hypomagnesemia-causing drugs [57, 63, 83]. The gold standard of treatment for PPI-induced hypomagnesemia is the withdrawal of PPIs. Within days after termination of PPI therapy, serum Mg^2+^ levels reach a normal level in most patients. In contrast, oral Mg^2+^ supplementation does not improve serum Mg^2+^ levels even at high doses. A treatment approach that has been gaining attention over the last years is the use of prebiotics. Inulin fibers have been demonstrated to increase serum Mg^2+^ levels in PPI users [20]. To what extent this treatment is of benefit for patients with other causes of hypomagnesemia remains to be determined in future research.

In recent years, sodium-glucose cotransporter 2 (SGLT2) inhibitors have gained increasing attention as potential treatment opportunity for hypomagnesemia. SGLT2 is expressed in the PT, where it is responsible for the reabsorption of glucose. Large clinical trials examining the efficacy of SGLT2 inhibitors for treatment of diabetes or cardiovascular defects, concomitantly demonstrated increased serum Mg^2+^ levels [84, 85]. One case even showed that treatment with SGLT2 inhibitors were more efficient in replenishing Mg^2+^ levels compared with oral Mg^2+^ supplementation [86].

Recently, several case reports have been published in which non-diabetic individuals were treated with SGLT2 inhibitors, resulting in significantly increased serum Mg^2+^ levels [85, 87]. As a result, the patients were able to decrease the dosage of their Mg^2+^ supplementation. This provides first evidence that SGLT2 inhibitors are not only of use in diabetic patients but also in hypomagnesemia patients with normal glucose metabolism. However, clinical trials need to be performed to confirm this effect.

The mechanism of action that explains the improved Mg^2+^ reabsorption in response to treatment with SGLT2 inhibitors has not been elucidated yet. It has been hypothesized that the increased Na^+^ content in the pre-urine leads to increased activity of NKCC2 in the TAL. Consequently, paracellular Mg^2+^ reabsorption would increase due to an increased transepithelial voltage potential. However, animal studies showed unchanged NKCC2 expression upon SGLT2 inhibition [88]. Interestingly, one study showed increased TRPM6 activity in Sprague–Dawley rats treated with the SGLT2 inhibitor dapagliflozin, suggesting that the increased Mg^2+^ reabsorption in the DCT explains the higher serum Mg^2+^ level [89]. The mechanism by which dapagliflozin increases TRPM6 activity is subject to future studies.

## CONCLUSION

To conclude, Mg^2+^ is essential for the physiological function of the human body and is tightly regulated by an interplay between intestinal Mg^2+^ absorption and renal Mg^2+^ excretion. Hypomagnesemia can either be genetic, acquired, or drug-induced by drugs such as PPIs, immunosuppressive drugs and diuretics. It often goes along with other comorbidities such as diabetes, metabolic syndrome or age. The first line of treatment for these patients is Mg^2+^ supplementation to increase serum Mg^2+^ levels; as of the time of writing there are no cures for the underlying causes of hypomagnesemia. SGLT2 inhibitors, however, shows promising effects in clinical case studies to increase serum Mg^2+^ levels in diabetic patients with hypomagnesemia.

## Data Availability

No new data were generated or analyzed for this manuscript.
